# Sensitivity and Specificity of Reflectance Confocal Microscopy and Dermoscopy in Assessing Vitiligo Staging

**DOI:** 10.1111/jocd.70892

**Published:** 2026-05-05

**Authors:** Jiyuan Wu, Wenjing Liu, Miaomiao Sun, Qian Jiang, Hongying Chen, Liuqing Chen

**Affiliations:** ^1^ Department of Dermatology Wuhan No. 1 Hospital Wuhan China; ^2^ Hubei Province and Key Laboratory of Skin Infection and Immunity Wuhan China; ^3^ Hubei University of Chinese Medicine Wuhan China; ^4^ Department of Dermatology Xiangyang Hospital of Traditional Chinese Medicine Xiangyang China

**Keywords:** dermoscopy, diagnosis, reflectance confocal microscopy, vitiligo, vitiligo staging

## Abstract

**Background:**

Accurate staging of vitiligo is essential for effective treatment. Current staging primarily relies on clinical manifestations, patients' subjective reports, and physicians' assessments, which may introduce inaccuracies. Reflectance confocal microscopy (RCM) and dermoscopy are auxiliary diagnostic tools in dermatology that support the diagnosis and differentiation of vitiligo; they also assist in staging and evaluating therapeutic efficacy.

**Aims:**

This study aimed to compare the sensitivity and specificity of RCM and dermoscopy in vitiligo staging, providing a more reliable reference for clinical application.

**Methods:**

A retrospective review was conducted of clinical and imaging data from 218 patients with vitiligo who attended the Department of Dermatology at Wuhan No. 1 Hospital between December 2023 and November 2024. Clinical assessment methods—clinical features, Vitiligo Disease Activity score, Wood's lamp examination, and Koebner phenomenon—served as the reference standard for staging. Blinded evaluators independently conducted staging using RCM and dermoscopy. Each modality's sensitivity and specificity were calculated.

**Results:**

Staging results based on RCM and dermoscopy showed strong concordance with clinical evaluation; Kappa values were 0.74 and 0.718, respectively (*p* < 0.01). The positive percent agreement and negative percent agreement of RCM staging with clinical assessment were 94.16% and 78.12%, respectively; those metrics for dermoscopy were 87.01% and 89.06%. RCM demonstrated significantly higher sensitivity than dermoscopy (*p* = 0.013), whereas dermoscopy showed significantly higher specificity than RCM (*p* = 0.039).

**Conclusions:**

RCM and dermoscopy provide significant clinical value in vitiligo staging. RCM shows higher positive percent agreement for detecting progressive disease, whereas dermoscopy shows higher negative percent agreement for confirming stable disease. These tools offer an objective foundation for assessment and support clinical decision‐making.

## 1 Introduction

Vitiligo is a common acquired hypopigmentation disorder characterized by circumscribed depigmented macules or patches on the skin and mucous membranes. The condition may present at any age and affect any cutaneous site; its estimated global prevalence is between 0.5% and 2% [1]. Current evidence suggests that the pathogenesis primarily involves autoimmune‐mediated, selective destruction of melanocytes by autoreactive cytotoxic T lymphocytes [2]. Lesions frequently appear on sun‐exposed areas and follow a chronic course. Although vitiligo does not directly impair physical health, it imposes a substantial psychosocial burden, affecting patients' daily life and overall well‐being. Treatment strategies vary according to disease stage [3]. In the progressive phase, first‐line options typically include systemic and/or topical glucocorticoids and Janus kinase inhibitors, often in combination with low‐dose ultraviolet phototherapy. In contrast, stable vitiligo requires a comprehensive therapeutic plan, which may involve systemic therapies, topical agents, phototherapy, surgical procedures (such as transplantation), laser treatment (e.g., fractional carbon dioxide laser), and potentially traditional Chinese medicine, all tailored to individual treatment goals. Therefore, early diagnosis combined with accurate staging and classification is essential to optimize therapeutic outcomes.

Several diagnostic tools are utilized for vitiligo, including physical examination by dermatologists, the Vitiligo Disease Activity (VIDA) score, the Vitiligo Area Scoring Index, the point‐counting method, and Wood's lamp examination [4]. Among these tools, the VIDA score, characteristic clinical features, the Koebner phenomenon, and Wood's lamp examination are most commonly used in clinical settings [5]. The use of these methodologies for the diagnosis and staging of vitiligo is limited by multiple factors. Clinical features and VIDA scores are contingent on physician expertise and patient‐reported symptoms, both of which are inherently subjective. The reliability of Wood's lamp examination is affected by variations in skin color, darkroom conditions, and medication‐induced fluorescence. These factors can lead to false‐negative or false‐positive findings, which can result in inaccurate staging and compromise the effectiveness of subsequent treatment. Although skin biopsy is considered the gold standard for diagnosing cutaneous diseases, its invasive nature carries the risk of triggering the Koebner phenomenon (homologous reaction), which may adversely affect recovery.

With the rapid advancement of cutaneous imaging technologies, reflectance confocal microscopy (RCM) and dermoscopy have become essential ancillary diagnostic tools in dermatology [6, 7]. These modalities are particularly useful in assessing vitiligo disease activity [8, 9, 10, 11]. RCM provides high‐resolution, real‐time imaging that allows detailed visualization of epidermal and upper dermal structures, achieving resolution comparable to that of histopathology [12]. In contrast, dermoscopy offers a non‐invasive and convenient method with high patient compliance, enabling direct visualization of superficial cutaneous features such as the pigment network and perifollicular changes. Although previous studies have independently analyzed the roles of dermoscopy and RCM in assessing vitiligo staging, few have directly compared the two modalities in this context.

In this study, clinical data, RCM images, and dermoscopic images were collected from 218 patients with vitiligo. Through systematic characterization of imaging features across active and stable disease phases, we sought to validate the core hypothesis that RCM's in vivo, non‐invasive, cellular‐resolution imaging provides superior sensitivity over dermoscopy in detecting subtle inflammatory dynamics and melanocyte alterations, thereby improving the accuracy of disease activity assessment. Addressing a literature gap regarding head‐to‐head comparisons of these modalities for vitiligo activity evaluation, we conducted a quantitative analysis of their diagnostic performance, comparing sensitivity and specificity. Our results demonstrated a statistically significant sensitivity advantage for RCM in identifying active‐phase biomarkers. These findings support the clinical utility of RCM as an objective tool for disease activity assessment, with potential to advance standardized staging methodologies and inform individualized therapeutic strategies. Collectively, this study provides foundational evidence for the development of evidence‐based imaging criteria to optimize clinical decision‐making.

## 2 Materials and Methods

### 2.1 Patient Sources

Clinical data were collected from 218 consecutive patients with vitiligo who attended the Department of Dermatology, Wuhan No. 1 Hospital, between December 2023 and November 2024. For patients with multiple lesions, the lesion with the most typical clinical presentation was selected for analysis to avoid clustering bias. Thus, one lesion per patient was analyzed. The cohort included patients from various geographic regions of China and comprised 101 male individuals and 117 female individuals (age range: 1 to 77 years). To ensure representativeness and minimize site‐specific bias, lesions were consecutively selected from these patients, encompassing multiple anatomical sites including the face, trunk, and extremities. The same consensus‐based staging criteria were applied uniformly to all lesions regardless of location.

### 2.2 Inclusion Criteria

1. Diagnosis in accordance with the Diagnosis and Treatment Standards for Melasma and Vitiligo (2010 Edition) [5], developed by the Pigmentology Group of the Chinese Society of Integrative Dermatology in 2010.
2. Lesions that had not received recent treatment.

### 2.3 Exclusion Criteria

1. Receipt of systemic therapy, glucocorticoids, immunosuppressants, phototherapy, or similar interventions within the past month.
2. History of severe cardiac, hepatic, or renal dysfunction.
3. History of photosensitive disorders.
4. Incomplete clinical or imaging data.

### 2.4 Clinical Information

Collected data included patient demographics (sex, age), lesion characteristics (site, region, stage, type), VIDA score, medical history, family history, and accompanying symptoms (e.g., pruritus, halo nevus, and poliosis/leukotrichia), as well as documentation of the Koebner phenomenon. Lesions were documented using clinical photographs under natural light, RCM images, and dermoscopic images.

### 2.5 Instruments and Methods

#### 2.5.1 RCM Examination

RCM was performed using the VivaScope 1500 system (Lucid Technologies, USA), which utilizes an 830 nm laser (0–16 mW power) and a 30× water immersion objective to provide 2‐μm lateral and 1.6‐μm axial resolution at scanning depths of 0–250 μm. Target lesions were cleansed before imaging; distilled water served as the immersion medium between the skin and adhesive ring. Medical ultrasound coupling gel was used as the interface between the objective lens and adhesive ring. The probe was positioned perpendicular to the lesion surface, and vertical scanning was conducted sequentially from the stratum corneum to the superficial dermis. En face (horizontal section) images were acquired to evaluate (1) epidermal pigment distribution, (2) basal cell layer integrity, and (3) the presence of inflammatory or dendritic cells.

#### 2.5.2 Dermoscopy

Dermoscopy was performed using the Medicam 800 workstation (FotoFinder Systems GmbH, Germany). Patients were positioned either seated or supine, with the lesion area fully exposed. Initial panoramic images were acquired at 10× magnification using cross‐polarized light mode and aligned with the device's digital markers. Non‐polarized mode was then selected, and the lens surface was disinfected with 75% ethanol. A continuous optical coupling interface was established by evenly applying distilled water or medical ultrasound gel to the lesion surface to reduce Fresnel reflectance. The probe was positioned perpendicularly to the skin at 40× magnification; uniform moderate pressure was applied to eliminate air bubbles while avoiding compression artifacts that could distort vascular structures. Focus was optimized; images were then acquired to evaluate pigment network architecture, vascular morphology, and other relevant microscopic features.

#### 2.5.3 Imaging Evaluation and Quality Control

RCM and dermoscopic images were assessed independently by two board‐certified dermatologists specialized in cutaneous imaging, each with over 5 years of dedicated experience in interpreting these modalities for pigmentary disorders. To ensure unbiased assessment, a strict blinding protocol was implemented: (1) the imaging evaluators were blinded to all clinical information, including the clinical staging results (VIDA score, Wood's lamp findings) and patient identity; and (2) they were also blinded to the results of the alternative imaging modality (i.e., RCM evaluators had no access to dermoscopic images of the same lesion, and vice versa). Interobserver reliability between the two imaging evaluators was calculated using Cohen's Kappa statistic.

### 2.6 Statistical Analyses

The positive percent agreement and negative percent agreement of the dermoscopy and RCM assessments were calculated using clinical assessment (VIDA score, Wood's lamp, Koebner phenomenon, and morphology) as the reference standard. Accounting for the paired nature of the data, differences in positive percent agreement and negative percent agreement between the RCM and dermoscopy were assessed using McNemar's test. The area under the receiver operating characteristic curve (AUC) was compared using DeLong's test. The Kappa statistic was used to evaluate agreement. Data were coded and entered using SPSS version 27.0 (IBM Corp.). The power of the current sample size was calculated using PASS 15 software (NCSS, Kaysville, Utah, USA). *p*‐values < 0.05 were considered statistically significant.

### 2.7 Methodology for Staging Judgments

To ensure systematic and reproducible staging, we employed standardized assessment scales for clinical evaluation, RCM, and dermoscopy. Each modality utilized a defined set of criteria, with staging determined by the composite score or the presence of key features as outlined below:

#### 2.7.1 Clinical Staging for Vitiligo

Clinical staging was performed by dermatologists based on a composite assessment scale derived from the 2021 consensus [3]. A patient was classified as Progressive Stage if they met one or more of the criteria (Table 1).

**TABLE 1:** Clinical diagnostic criteria for advanced stage.

Criterion	Description	Score/indicator
VIDA score	Based on timing of new lesions/expansion: +4 (≤ 6 weeks), +3 (≤ 3 months), +2 (≤ 6 months), +1 (≤ 1 year)	Score > +1
Active signs	Ill‐defined borders, inflammatory halo, trichrome/confetti lesions	Present
Koebner phenomenon	New lesions at sites of trauma within past year	Positive
Wood's lamp	Grayish‐white hue, blurred borders, illuminated area>visible area	Positive

A patient was classified as Stable Stage if they met one or more of the criteria (Table 2).

**TABLE 2:** Clinical criteria for the stable phase.

Criterion	Description	Score/indicator
VIDA score	0 (stable > 1 year) or −1 (stable ≥ 1 year with repigmentation)	Score ≤ 0
Lesion morphology	Porcelain‐white color, sharp borders, marginal hyperpigmentation	Present
Koebner phenomenon	Absence of isomorphic response for ≥ 1 year	Negative
Wood's lamp	Bright‐white hue, sharp borders, illuminated area≤visible area	Positive

#### 2.7.2 RCM Staging Scale

RCM images were evaluated by imaging physicians using a semi‐quantitative activity scale adapted from Le et al. [13]. Three key features were scored, and the total score determined the stage (Table 3). The total score derived from these features was used to determine the disease stage: a total score of ≥ +2 was classified as the progressive stage, while a score of ≤ 0 indicated the stable stage.

**TABLE 3:** RCM mobility assessment scale.

Assessment feature	Findings	Score
Pigmentation of lesional skin	Residual pigment	+1
	Complete pigment loss	−1
Basal layer pigment ring integrity	Uneven or partial loss	+1
	Complete absence	−1
Inflammatory cell infiltration	Melanophagocytes at lesion edge	+2
	Lymphocytes	+1
	No inflammatory cells	−1

#### 2.7.3 Dermoscopic Staging [14]

Dermoscopic features indicative of the progressive stage include blurred lesion margins, absence of perifocal pigmentation, disrupted pigment reticulation, a creamy or porcelain‐white background, satellite phenomenon, tapioca sago‐like structures, and either the micro‐Koebner or comet tail phenomenon. In contrast, clear lesion margins and perifocal hyperpigmentation indicate the stable stage.

## 3 Results

### 3.1 RCM and Dermoscopic Imaging Features in Patients With Vitiligo at Different Stages

**FIGURE 1:**
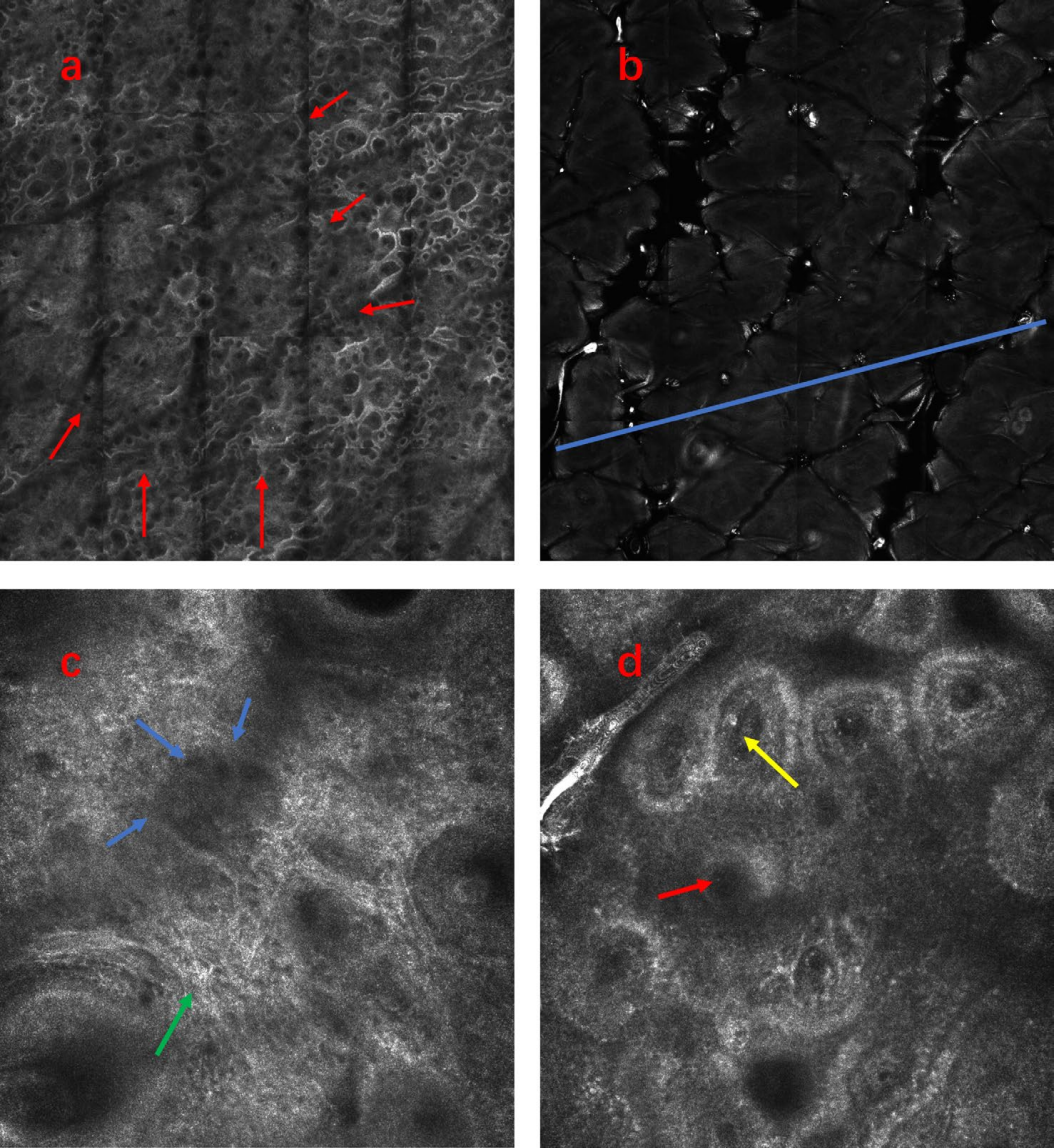
RCM features of patients with vitiligo. (a) Blurred lesion border (red arrow); (b) clear border; (c) the basal layer dritic melanocytes (green arrow), the basal layer exhibits an absence of pigment rings (blue arrow); (d) derin complete pigment ring (red arrow), inflammatory cell infiltration (yellow arrow).

**FIGURE 2:**
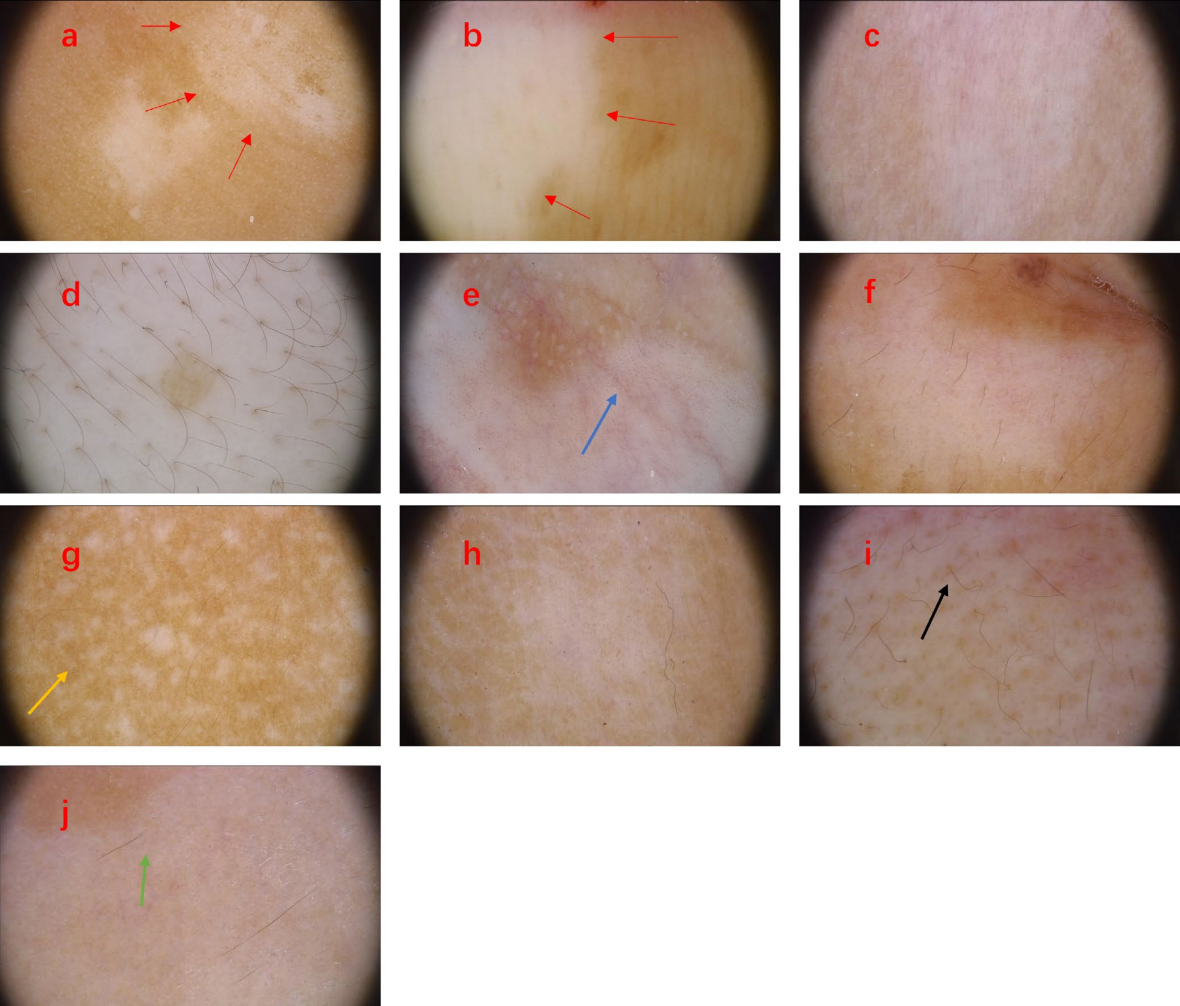
Dermoscopic features of patients with vitiligo. (a) Blurred lesion border (red arrow); (b) clear lesion border (red arrow); (c) white creamy background; (d) porcelain white background; (e) telangiectasia (blue arrow); (f) hyperpigmentation surrounding the lesions; (g) satellite lesions (yellow arrow); (h) faint pigment network; (i) perifollicular pigmentation (black arrow); (i) perifollicular depigmentation (green arrow).

Under RCM, the basal layer of the lesion may exhibit an incomplete or entirely absent pigment ring, accompanied by infiltration of inflammatory cells or melanophagocytes. Lesion borders may appear either clear or blurred (Figure 1). Dermoscopic features include clear or blurred lesion borders, a creamy or porcelain‐white background, residual or absent perifollicular pigmentation, leukotrichia, telangiectasia, and perifocal hyperpigmentation, along with distinctive findings such as the tapioca sago appearance and satellite lesions (Figure 2).

### 3.2 Positive and Negative Percent Agreement of RCM and Dermoscopic With Clinical Staging for Vitiligo

**FIGURE 3:**
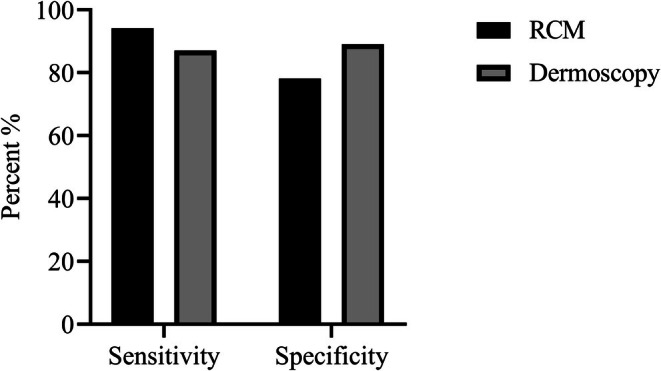
Comparison of sensitivity and specificity of RCM and Dermoscopy in the staging of vitiligo.

As shown in Table 4, there was significant consistency between RCM staging and clinical evaluation (kappa = 0.740, *p < 0.001)*, RCM staging and dermatoscopic staging (kappa = 0.724, *p < 0.001)*, and dermatoscopic staging and clinical staging (kappa = 0.718, *p < 0.001)*. The clinical assessment method—comprising clinical characteristics, the Vitiligo Disease Activity (VIDA) score, Wood's lamp examination, and the Koebner phenomenon—served as the reference standard. Although this method has minor limitations, it is currently recognized as the diagnostic staging standard in the 2021 consensus on vitiligo diagnosis and treatment. The positive and negative percent agreements of RCM and dermoscopic assessments are presented in Table 5 and Figure 3. Specifically, RCM staging yielded sensitivity and specificity values of 94.16% and 78.12%, respectively, while dermoscopic staging achieved values of 87.01% and 89.06%. The positive percent agreement of RCM was significantly higher than that of dermoscopy (*p* = 0.013, McNemar's test), whereas the negative percent agreement of dermoscopy was significantly higher than that of RCM (*p* = 0.039, McNemar's test).

**TABLE 4:** Comparison of concordance among RCM, dermoscopy, and clinical vitiligo disease activity staging.

	Progressive stage	Stable stage	Total cases	Kappa	*p*
Clinical evaluation	154	64	218	0.740^a^	< 0.001
RCM	159	59	218	0.724^b^	< 0.001
Dermoscopy	141	77	218	0.718^c^	< 0.001

Abbreviation: RCM, reflectance confocal microscopy.

^a^ RCM was significantly consistent with Clinical evaluation (*p* < 0.001).

^b^ RCM was significantly consistent with Dermoscopy (*p* < 0.001).

^c^ Dermoscopy was significantly consistent with Clinical evaluation (*p* < 0.001).

**TABLE 5:** Positive and negative percent agreement of RCM and dermoscopic assessments with clinical staging.

	RCM (95% CI)	Dermoscopy (95% CI)	*p*
Positive percent agreement (%)	94.16 (91.04–97.27)	87.01 (82.55–91.48)	0.013
Negative percent agreement (%)	78.12 (72.64–83.61)	89.06 (84.92–93.21)	0.039

Abbreviation: CI, confidence interval.

**FIGURE 4:**
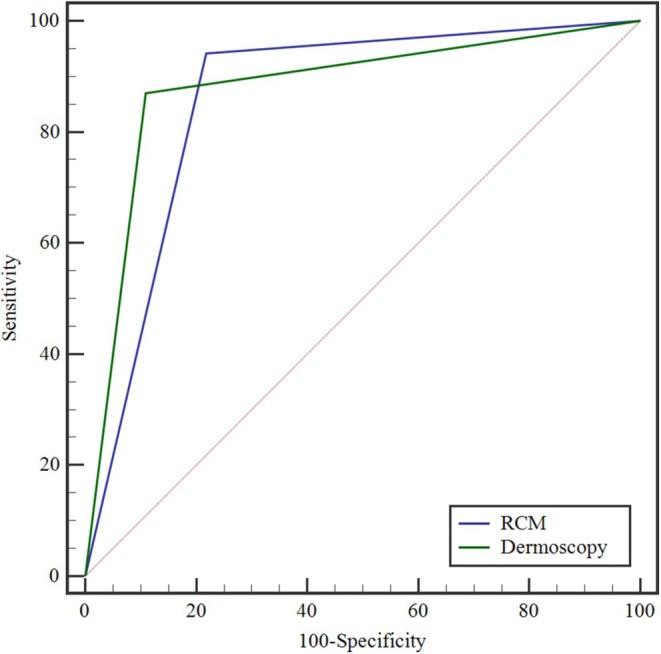
ROC Curve.

The analysis was executed through Pass 15 software for Tests for One‐Sample Sensitivity and Specificity. Post hoc power analysis assuming one lesion per patient indicated adequate statistical power for the primary comparisons. Furthermore, as demonstrated in Figure 4, the ROC curves revealed an AUC of 0.861 (95% CI: 0.808–0.904) for RCM and 0.880 (95% CI: 0.830–0.920) for dermoscopy. A subsequent analysis using DeLong's test revealed no statistically significant difference between the AUC values (*p* = 0.468).

## 4 Discussion

RCM enables non‐invasive, real‐time imaging at the cellular level in a manner that permits dynamic, repeated assessments [15]. In the RCM imaging data collected from 218 patients with vitiligo in this study, partial loss of the basal cell pigment ring, infiltration of lymphocytes or melanophagocytes, and indistinct borders between lesions and surrounding normal skin were observed at the progressive stage of disease. In contrast, lesions in patients with stable vitiligo exhibited complete absence of the basal cell pigment ring, absence of inflammatory cell infiltration, and presence of dendritic cells. These results are consistent with findings from multiple domestic and international studies [13, 16, 17].

Dermoscopy, a non‐invasive diagnostic tool, plays a critical role in the diagnosis of various skin conditions, including tumors, inflammatory disorders, and infections [18, 19, 20]. In this study, analysis of 218 dermoscopic images revealed several characteristic features in lesions from patients at the progressive stage, including blurred margins, creamy‐white backgrounds, perifollicular pigmentation, vasodilatation, and, in some cases, the tapioca sago and satellite phenomena described by Fu Chao [14]. In contrast, lesions in the stable stage displayed clear borders, porcelain‐white backgrounds, perifollicular depigmentation, leukotrichia, and perilesional or marginal hyperpigmentation. These observations are consistent with prior findings [8, 21, 22, 23, 24].

This study examined the differences between RCM and dermoscopy in the assessment of vitiligo staging. Experts worldwide have emphasized the importance of early disease detection, accurate staging, and formulation of treatment strategies tailored to each disease phase [2, 25]. Although both RCM and dermoscopy have demonstrated utility in vitiligo staging [26, 27], few studies have directly compared their performance. Clarification of differences between these two techniques and accurate determination of disease stage through scientific and quantitative methods will support the development of individualized treatment plans and enhance patient outcomes. Notably, the present findings extend previous single‐modality studies. Li et al. utilized RCM to evaluate 125 vitiligo patients and reported that loss of pigment‐ring integrity, unclear borders, and the presence of highly refractile inflammatory cells at the lesion edge were indicative of active disease [13]. Our RCM observations are consistent with those features, but we further quantified the diagnostic performance, demonstrating a positive percent agreement of 94.16% for detecting active vitiligo, a metric not previously reported in their work. Similarly, Nirmal et al. established the BPLFeoSK dermoscopic criteria for stability, highlighting features such as sharp borders, absent/reticulate pigment network, and absence of satellite lesions or micro‐Koebner phenomenon [27]. Our dermoscopic results align with those criteria, yet we provide a head‐to‐head comparison with RCM, showing that dermoscopy achieved higher negative percent agreement (89.06% vs. 78.12%, *p* = 0.039) for stable‐stage identification. By directly comparing both modalities in the same cohort, this study not only corroborates key observations from earlier single‐technique reports but also offers new evidence on their relative strengths, RCM being more sensitive for detecting active disease and dermoscopy more specific for confirming stability. This comparative perspective aids clinicians in selecting the most appropriate tool based on the clinical question, thereby advancing beyond the isolated use of either technique.

RCM enables observation of cellular structures from the epidermis to the superficial dermis at a microscopic level and permits identification of dynamic cellular changes through three‐dimensional imaging. This technique allows the detection of subtle pathological changes associated with vitiligo staging and can substantially reduce the rate of missed diagnoses [28]. In addition to its utility in staging, RCM also serves as a valuable tool for monitoring therapeutic response. A recent study on combined treatment with narrowband ultraviolet B and topical piperine in localized vitiligo demonstrated that morphological improvements, including a reduction in irregular honeycomb patterns and non‐pigmented papillae along with an increase in dendritic cells and blood vessels, were clearly observable via RCM [29]. These microstructural changes correlated with clinical repigmentation, underscoring the role of RCM in objectively evaluating treatment efficacy and reinforcing its potential in guiding and adjusting therapeutic strategies over time. Despite its advantages, RCM has important limitations in terms of clinical applications. As demonstrated by Wu and colleagues, variations in the scanned location of vitiligo lesions may result in disparate staging outcomes. The lesion center provides greater sensitivity for identifying disease progression, whereas the surrounding normal skin offers the highest specificity for identifying stable vitiligo [30]. Dermoscopy constitutes an additional non‐invasive imaging modality. Although its visualization is limited to the epidermis and papillary dermis, dermoscopy offers considerable advantages in terms of accessibility and clinical utility. It can detect early pigmented islands, assess treatment response in pigmented dermatoses, and identify subtle changes not visible to the naked eye. These capabilities contribute to improved patient compliance and enhanced therapeutic outcomes [31]. Our cohort included lesions from varied anatomical sites (face, trunk, extremities). The reported sensitivity and specificity, therefore, represent an aggregate performance across body regions, enhancing the clinical generalizability of our findings. While site‐specific variations in imaging features exist, the diagnostic criteria used are based on morphological features (e.g., border definition, pigment pattern, cellular infiltration) that are applicable across sites. The consistent staging accuracy observed across different locations in our study supports the robustness of these consensus criteria for clinical use.

A comparative analysis of the two imaging modalities, RCM and dermoscopy, in the assessment of vitiligo staging facilitates selection of appropriate diagnostic and therapeutic tools, thereby supporting the development of more precise, individualized treatment plans. This approach may improve patient prognosis, enhance compliance, and optimize treatment efficacy. In the present study, RCM demonstrated a statistically significant advantage in identifying the progressive stage of vitiligo, with higher positive percent agreement (*p* < 0.05). This makes it particularly suitable for evaluation of patients with early or active disease and enables detection of subtle changes at the borders of depigmented lesions, thus reducing the likelihood of missed diagnoses. In contrast, dermoscopy exhibited higher negative percent agreement for diagnosing vitiligo in the stable stage (*p* < 0.05), with clear visualization of residual pigment islands inside depigmented areas and detailed assessment of capillary morphology, reducing the risk of misdiagnosis in stable‐stage patients. The translation of these diagnostic performance metrics into clinical practice necessitates a critical appraisal of pragmatic implementation barriers. RCM, while providing near‐histological resolution, is a capital‐intensive technology with significant operational costs, and its image interpretation requires specialized training, constraining its dissemination to tertiary referral centers. Dermoscopy, validated as a cost‐effective and rapid bedside tool with a comparatively shorter learning curve, offers greater potential for widespread adoption in primary and secondary care settings. This accessibility differential informs a staged, precision medicine approach to vitiligo management. The superior sensitivity of RCM renders it a pivotal tool for resolving diagnostic uncertainty in early or subclinical progression. The detection of subtle inflammatory infiltrates or focal melanocyte loss at the advancing edge via RCM may provide objective information that supports clinical judgment when disease activity remains equivocal on clinical and dermoscopic examination. Future studies incorporating treatment outcomes are warranted to validate the predictive value of these imaging features. Conversely, the high specificity of dermoscopy in identifying stable disease, characterized by well‐defined depigmentation and perifollicular pigment loss, correlates with a higher likelihood of success for surgical interventions such as melanocyte‐keratinocyte transplantation. Thus, dermoscopy can reliably guide the transition from medical to surgical treatment modalities, optimizing resource allocation. An integrative imaging protocol, leveraging RCM for initial activity assessment in complex cases and dermoscopy for monitoring stability and treatment response, may establish a new standard for evidence‐based, individualized therapeutic stratification in vitiligo.

Given these differences in diagnostic performance, a staging‐oriented imaging selection strategy is recommended for clinical application. When patients present with progressive features—such as indistinct lesion margins, inflammatory vitiligo, or trichrome or papery‐white patches—RCM is advisable due to its capacity for dynamic monitoring and real‐time assessment of melanocyte activity. For patients in the stable stage, characterized by sharp lesion borders and porcelain‐white patches, dermoscopy is the preferred modality because it facilitates differential diagnosis through detailed analysis of the pigment network and vascular patterns. In cases where staging characteristics remain ambiguous, a combined approach using both RCM and dermoscopy is recommended. This method involves initial screening with RCM to detect early signs of progression, followed by confirmation of disease stability via dermoscopic evaluation. When patients have limited financial resources or require rapid screening, RCM may be prioritized for staging assessment in combination with clinical features (e.g., Koebner phenomenon, leukotrichia), and supplemented by dermoscopy when necessary. The integrated use of imaging technologies provides complementary advantages, enhancing the accuracy of staging assessment and improving the allocation of medical resources. This dual‐modality approach offers robust imaging evidence to inform the selection of individualized treatment strategies, including phototherapy, pharmacologic interventions, or surgical procedures. In this study, a composite clinical assessment incorporating the VIDA score, Wood's lamp examination, and evaluation of the Koebner phenomenon served as the reference standard for vitiligo staging. This approach aligns with current consensus guidelines and represents the most feasible and clinically established method, particularly given the impracticality and risk of inducing the Koebner phenomenon associated with skin biopsy in active disease. However, it is important to acknowledge that clinician‐reported outcome measures, while essential, inherently possess a degree of subjectivity, and their measurement properties require continuous evaluation [32]. This lack of a perfect, objective gold standard is a common methodological challenge in dermatology research, as highlighted in studies validating diagnostic criteria where expert consensus often serves as the benchmark in the absence of histopathology [33]. Consequently, any potential misclassification by the clinical reference standard could theoretically influence the calculated agreement estimates of the imaging modalities under investigation. To mitigate this inherent subjectivity and strengthen the reliability of our reference standard, all clinical evaluations in this study were independently performed by two board‐certified dermatologists, with imaging assessments conducted by specialized imaging physicians blinded to the clinical findings. This rigorous, multi‐rater design was implemented to maximize diagnostic consistency and minimize individual assessment bias.

This study had the following limitations: (1) This was a single‐center study with a moderately sized cohort. Although lesions from multiple anatomical sites were included, future multi‐center studies with larger samples could further validate the generalizability of our findings across different skin types and regional practices. (2) Imaging interpretation presents a dual challenge. First, there are currently no standardized quantitative criteria for the analysis of RCM and dermoscopic images. Second, image interpretation may be influenced by the subjective judgment of physicians with diverse levels of experience. Thus, there is a need to develop guidelines or consensus standards for skin imaging. (3) The definition of progressive disease in this study required meeting any one of several clinical criteria, which resulted in a relatively high proportion of progressive cases (71%) in the cohort. This may introduce spectrum bias and could influence agreement estimates. To provide additional clinical context, positive likelihood ratios (LR+) and negative likelihood ratios (LR–) were calculated. For RCM, LR+ was 4.30 and LR– was 0.07; for dermoscopy, LR+ was 7.95 and LR– was 0.15. These values suggest that both modalities offer meaningful information to support clinical staging, although they should be interpreted in light of the pretest probability and the absence of a true gold standard. (4) Additionally, the present study is limited in scope because it compares only the progressive and stable stages. It does not include assessment of the rapid progression or repigmentation phases. Future research will address these phases through expanded sample collection and longitudinal imaging analysis.

In the current protocol, clinical staging was first determined by attending physicians using clinical assessment methods, including clinical characteristics, the VIDA score, Wood's lamp examination, and the Koebner phenomenon. Subsequently, dermoscopic and RCM examinations were performed by a professional imaging technologist blinded to the clinical staging results.

## Author Contributions

All authors have read and approved the final manuscript. Jiyuan Wu, Wenjing Liu, and Miaomiao Sun performed the research. Jiyuan Wu, Wenjing Liu, and Miaomiao Sun designed the study. Liuqing Chen, Qian Jiang, and Hongying Chen contributed essential reagents or tools. Jiyuan Wu and Wenjing Liu analyzed the data. Jiyuan Wu and Wenjing Liu wrote the paper.

## Funding

This work was supported by Joint Fund Project of Hubei Provincial Administration of Traditional Chinese Medicine Joint Fund Project (Grant No. ZY2025L018).

## Ethics Statement

We confirm that the ethical policies of the journal, as noted on the journal's author guidelines page, have been adhered to, and the appropriate ethical review committee approval has been received. The Medical Ethical Committee of the Wuhan No. 1 Hospital (MEC‐2024‐82).

## Conflicts of Interest

The authors declare no conflicts of interest.

## Data Availability

Research data are not shared.
